# The Effect of the Anisotropy of Single Crystal Silicon on the Frequency Split of Vibrating Ring Gyroscopes

**DOI:** 10.3390/mi10020126

**Published:** 2019-02-14

**Authors:** Zhengcheng Qin, Yang Gao, Jia Jia, Xukai Ding, Libin Huang, Hongsheng Li

**Affiliations:** 1 School of Instrument Science and Engineering, Southeast University, Nanjing 210096, China; 230189281@seu.edu.cn (Z.Q.); 230169207@seu.edu.cn (J.J.); dingxukai@126.com (X.D.); huanglibin@seu.edu.cn (L.H.); 2 Key Laboratory of Micro-Inertial Instruments and Advanced Navigation Technology, Ministry of Education, Nanjing 210096, China; 3 Artificial Intelligence Institute of Industrial Technology, Nanjing Institute of Technology, Nanjing 211167, China; ygao@njit.edu.cn

**Keywords:** single crystal silicon, anisotropy, vibrating ring gyroscope, frequency split

## Abstract

This paper analyzes the effect of the anisotropy of single crystal silicon on the frequency split of the vibrating ring gyroscope, operated in the n=2 wineglass mode. Firstly, the elastic properties including elastic matrices and orthotropic elasticity values of (100) and (111) silicon wafers were calculated using the direction cosines of transformed coordinate systems. The (111) wafer was found to be in-plane isotropic. Then, the frequency splits of the n=2 mode ring gyroscopes of two wafers were simulated using the calculated elastic properties. The simulation results show that the frequency split of the (100) ring gyroscope is far larger than that of the (111) ring gyroscope. Finally, experimental verifications were carried out on the micro-gyroscopes fabricated using deep dry silicon on glass technology. The experimental results are sufficiently in agreement with those of the simulation. Although the single crystal silicon is anisotropic, all the results show that compared with the (100) ring gyroscope, the frequency split of the ring gyroscope fabricated using the (111) wafer is less affected by the crystal direction, which demonstrates that the (111) wafer is more suitable for use in silicon ring gyroscopes as it is possible to get a lower frequency split.

## 1. Introduction

With the development of the microelectromechanical systems (MEMS) technology, MEMS inertial sensors have been widely adopted into many fields, such as aerospace, vehicle navigation, and consumer electronic products including smartphones, tablets, and wearable sensors [1]. One of the most common MEMS sensors is the vibrating gyroscope. Compared with the traditional gyroscope, the MEMS gyroscope has many advantages, including a smaller volume, lower power consumption, wider measurement range, and higher reliability. The vibrating ring gyroscope (VRG) is a kind of MEMS gyroscope that has the following merits over other similar types. Firstly, the VRG has better mechanical sensitivity characteristics as well as being less sensitive to environmental interferences such as shock and temperature variations. This is because of its symmetric structure and the equal resonant frequencies of the drive and sense modes [2]. In addition, the VRG has a wider bandwidth and full-scale range [3]. Moreover, the VRG can be used to measure the angle directly, which helps avoid the continuous accumulation of the test error during the process of the integration by measuring the angle rate [4].

At present, the high respect ratio VRG can be fabricated using single crystal silicon (SCS) because of the mature bulk silicon micromachining technique. The SCS is a very significant MEMS material, and it is often used to fabricate the MEMS resonator and substrate due to its excellent mechanical and electrical properties. However, the anisotropy of the SCS makes the elastic properties of the SCS vary with respect to the crystal orientations [5], which may influence the effective stiffness of the drive and sense modes of the VRG. As a result, resonant frequencies of the n=2 wineglass mode of the VRG are not always equal. The frequency mismatch can cause many problems, such as the angle-dependent bias and a decrease in sensitivity thus hindering the performance of the VRG [6]. So it is essential to analyze the relationship between elastic properties and crystal directions of the SCS during the process of designing the VRG. In order to avoid this trouble, some researchers prefer the n=3 mode rather than the n=2 mode, because the n=3 mode is inherently identical, which helps to eliminate the frequency mismatch induced by the anisotropic elastic properties of the SCS [7]. However, both the angular gain and the effective mass of the n=3 mode are smaller than those of the n=2 mode, and the sensitivity of the n=3 mode is lower [8,9]. Besides, resonant frequencies of the n=3 mode are higher, and the electrode arrangement is more complicated, which raises the difficulty of the circuit design and realization. For the reasons mentioned above, the n=2 mode of the SCS VRG is usually the ideal choice.

There are some theories about the relationship between the frequency split and the anisotropy of the SCS circular ring. For example, Hamilton’s principle was used to derive equations of the in-plane motion of the SCS circular ring, and the frequency split was explained by the conservation of averaged mechanical energy [10]. In addition, in-plane and out-of-plane resonant modes of the circular ring were solved by Lagrange’s equations, and the effect of anisotropy was considered in the strain energy formulation [5]. However, these theories derived from the perspective of the single ring were not concretely combined with a real SCS VRG or confirmed by experiments. The effects of the anisotropy of SCS on resonant frequencies of other resonators such as the cantilever and the disk were reported. For instance, the influence of crystallographic orientation on resonant frequencies of (100)-oriented resonators was investigated by a micromachined array consisting of 36 cantilevers in Reference [11]. In Reference [12], frequency splits of (100) and (111) disk resonators with different modes were examined and compared experimentally. Besides, an analytical formulation of frequency splits of SCS disk resonators was represented by taking material anisotropy into account [13]. Though some researchers prefer the (111) SCS to fabricate n=2 mode ring and disk gyroscopes because of the uniform material properties of this material, the reason for this was not analyzed and explained systematically in these articles [14,15]. Therefore, there is still a lack of research about how the anisotropy influences the frequency characteristics of the SCS VRG.

This paper aims to analyze the influence of the anisotropy of the SCS on the frequency split of the SCS VRG. The elastic properties of (100) and (111) wafers are analyzed, and the ring gyroscopes of these wafers are simulated, fabricated, and tested to prove that the (111) VRG has a tiny frequency split, so the (111) wafer is more suitable than the (100) wafer to make VRGs. This paper is organized as follows. Section 2 calculates the elastic matrices and orthotropic elasticity values of (100) and (111) wafers. In Section 3, (100) and (111) VRGs are simulated by finite element analysis. VRGs of different wafers are fabricated and tested in Section 4 to verify the theory and the simulation. Section 5 is the conclusion of this paper.

## 2. Anisotropic Elastic Properties of SCS

The linear proportional relationship between the strain ε and the stress σ of solids such as silicon is proved by the Hooke’s law,

(1) $$\varepsilon_{ij}=S_{ijkl}\sigma_{kl},\sigma_{ij}=C_{ijkl}\varepsilon_{kl}$$

where *S* and *C* are the compliance tensor and the stiffness tensor, respectively; i,j,k,l=x,y,z in the rectangular coordinate. The fourth rank stiffness or compliance tensor can be simplified as a 6×6 matrix with 21 independent constants due to the symmetry of the crystal. Meanwhile, subscripts with four elements in Equation (1) can be simplified to two elements: xx→1, yy→2, zz→3, yz and zy→4, xz and zx→5, xy and yx→6 [16].

The anisotropy of the SCS is reflected in the variety of physical properties related to crystal orientations. Each crystal plane or crystal orientation can be identified by Miller indices and written in the form of (*ijk*) or [*ijk*], respectively [17]. Three axes *X* ([100]), *Y* ([010]), *Z* ([001]) of the default coordinate system based on the right-hand rule lay along three baselines of the crystal cubic, so corresponding default stiffness and compliance matrices along the crystal orientations according to Hooke’s law can be represented as

(2) $$C=\left[\begin{array}{cccccc} c_{11} & c_{12} & c_{12} & 0 & 0 & 0\\ c_{12} & c_{11} & c_{12} & 0 & 0 & 0\\ c_{12} & c_{12} & c_{11} & 0 & 0 & 0\\ 0 & 0 & 0 & c_{44} & 0 & 0\\ 0 & 0 & 0 & 0 & c_{44} & 0\\ 0 & 0 & 0 & 0 & 0 & c_{44} \end{array}\right]=S^{-1}={\left[\begin{array}{cccccc} s_{11} & s_{12} & s_{12} & 0 & 0 & 0\\ s_{12} & s_{11} & s_{12} & 0 & 0 & 0\\ s_{12} & s_{12} & s_{11} & 0 & 0 & 0\\ 0 & 0 & 0 & s_{44} & 0 & 0\\ 0 & 0 & 0 & 0 & s_{44} & 0\\ 0 & 0 & 0 & 0 & 0 & s_{44} \end{array}\right]}^{-1}$$

where $c_{11}=1.657\times 10^{11}$ Pa, $c_{12}=0.639\times 10^{11}$ Pa, $c_{44}=0.796\times 10^{11}$ Pa, $s_{11}=0.768\times 10^{-11}$ Pa${}^{-1}$, $s_{12}=-0.214\times 10^{-11}$ Pa${}^{-1}$, $s_{44}=1.26\times 10^{-11}$ Pa${}^{-1}$ at room temperature [18]. Next, anisotropic elasticity analyses of (100) and (111) silicon wafers are based on these two matrices.

**Figure 1:**
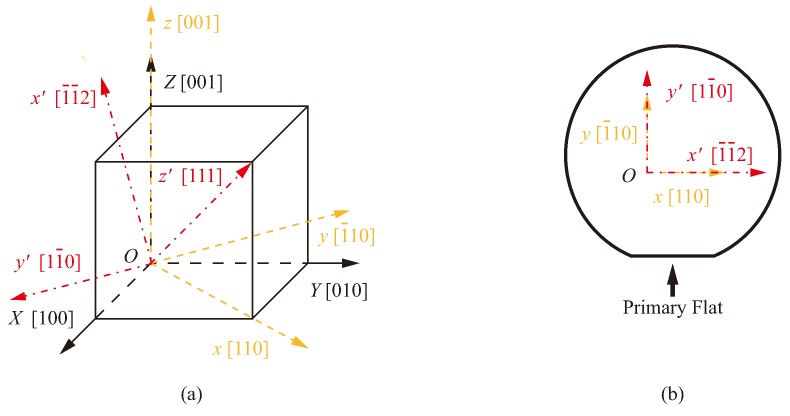
Coordinate systems and overhead views of the (100) and (111) silicon wafers: (**a**) Coordinate systems of two wafers, (**b**) Overhead views of two wafers.

Though the top surface of the (100) silicon wafer is perpendicular to the [100] direction, the actual coordinate system of the structure fabricated on the (100) silicon wafer is not consistent with the default coordinate system. The xyz coordinate system of the (100) wafer can be obtained by rotating the default coordinate system anti-clockwise through 45 degrees about the *Z*-axis [19]. So orthogonal axes (*x*, *y*, *z*) keep the same directions with three yellow dashed lines in Figure 1a. Figure 1b indicates that the primary flat of the (100) wafer is parallel to the [110] direction that is exactly the *x*-axis. For the (111) silicon wafer, the [111] orientation is vertical to the crystal surface of this wafer. Coordinate axes (x′, y′, z′) of the (111) wafer are represented by three red dot-dashed lines in Figure 1a successively, because coordinate axes of the (111) wafer shown in Figure 1a can be converted by rotating the default coordinate system twice. At first, the default coordinate system is rotated clockwise 135 degrees about the *Z*-axis, then through a clockwise angle of 54.7 degrees about the transformed *Y*-axis (i.e., y′-axis). The x′-axis in Figure 1b is parallel to the primary flat of the (111) wafer and perpendicular to the y′-axis which is the $\left[1\bar{1}1\right]$ direction.

After getting the coordinate systems of the (100) and (111) wafers, corresponding stiffness matrices and compliance matrices can be figured out using the method developed by Bond [16]. Direction cosines of each rotated axis should be firstly given, which are three cosines of angles between the rotated axis and three default axes (i.e., [100], [010], and [001]). Three terms *l*, *m*, and *n* are used to define direction cosines of an axis. Therefore, direction cosines of three axes of any rotated coordinate system can build a 3 × 3 matrix ***R*** to describe the transformation from the default coordinate system. Matrices ***R***s of coordinate systems of (100) and (111) wafers are presented as

(3) $$R_{100}=\left[\begin{array}{ccc} \frac{1}{\sqrt{2}} & \frac{1}{\sqrt{2}} & 0\\ \frac{-1}{\sqrt{2}} & \frac{1}{\sqrt{2}} & 0\\ 0 & 0 & 1 \end{array}\right],$$

(4) $$R_{111}=\left[\begin{array}{ccc} \frac{-1}{\sqrt{6}} & \frac{-1}{\sqrt{6}} & \frac{2}{\sqrt{6}}\\ \frac{1}{\sqrt{2}} & \frac{-1}{\sqrt{2}} & 0\\ \frac{1}{\sqrt{3}} & \frac{1}{\sqrt{3}} & \frac{1}{\sqrt{3}} \end{array}\right].$$

Then, stiffness matrices of (100) and (111) wafers can be obtained according to the method in Reference [16]. Because the compliance matrix is the inverse of the stiffness matrix, only the stiffness matrices of (100) and (111) wafers are calculated and shown as

(5) $$C_{100}=\left[\begin{array}{cccccc} 1.944 & 0.352 & 0.639 & 0 & 0 & 0\\ 0.352 & 1.944 & 0.639 & 0 & 0 & 0\\ 0.639 & 0.639 & 1.657 & 0 & 0 & 0\\ 0 & 0 & 0 & 0.796 & 0 & 0\\ 0 & 0 & 0 & 0 & 0.796 & 0\\ 0 & 0 & 0 & 0 & 0 & 0.509 \end{array}\right]\times 10^{11}\left(\mathrm{Pa}\right),$$

(6) $$C_{111}=\left[\begin{array}{cccccc} 1.9440 & 0.5433 & 0.4477 & 0 & -0.1353 & 0\\ 0.5433 & 1.9440 & 0.4477 & 0 & 0.1353 & 0\\ 0.4477 & 0.4477 & 2.0397 & 0 & 0 & 0\\ 0 & 0 & 0 & 0.6047 & 0 & 0.1353\\ -0.1353 & 0.1353 & 0 & 0 & 0.6047 & 0\\ 0 & 0 & 0 & 0.1353 & 0 & 0.7003 \end{array}\right]\times 10^{11}\left(\mathrm{Pa}\right).$$

Obtaining correct elastic properties of a wafer is the premise of achieving the correct modal simulation of a structure fabricated on this wafer. In the practical simulation process, apart from inputting the stiffness matrix or the compliance matrix, employing orthotropic elasticity values of the wafer can be used to simulate resonant frequencies of the VRG [19].

Orthotropic elasticity values which contain Young’s modulus *E*, Poisson ratio υ, and the shear modulus *G* also depend on the direction cosines of coordinate axes of the wafer. These values can be given as

(7) $$\frac{1}{E}=s_{11}-2\left(s_{11}-s_{12}-\frac{1}{2}s_{44}\right)\left(l^{2}m^{2}+m^{2}n^{2}+l^{2}n^{2}\right),$$

(8) $$\upsilon =-\frac{s_{12}+\left(s_{11}-s_{12}-\frac{1}{2}s_{44}\right)\left(l_{1}^{2}l_{2}^{2}+m_{1}^{2}m_{2}^{2}+n_{1}^{2}n_{2}^{2}\right)}{s_{11}-2\left(s_{11}-s_{12}-\frac{1}{2}s_{44}\right)\left(l_{1}^{2}m_{1}^{2}+m_{1}^{2}n_{1}^{2}+l_{1}^{2}n_{1}^{2}\right)},$$

(9) $$\frac{1}{G}=s_{44}+4\left(s_{11}-s_{12}-\frac{1}{2}s_{44}\right)\left(l_{1}^{2}l_{2}^{2}+m_{1}^{2}m_{2}^{2}+n_{1}^{2}n_{2}^{2}\right)$$

where ($l_{1}$, $m_{1}$, $n_{1}$) and ($l_{2}$, $m_{2}$, $n_{2}$) are two groups of direction cosines of two orthogonal directions with respect to the crystal axes [20,21].

In particular, direction cosines of any directions in the plane of the (111) wafer meet the conditions that l+m+n=0 and $l^{2}+m^{2}+n^{2}=1$. Therefore, the conclusion can be derived that $l^{2}m^{2}+l^{2}n^{2}+m^{2}n^{2}=1/4$, which means Young’s modulus in the plane of the (111) wafer is invariable. Further, another conclusion that $l_{1}^{2}l_{2}^{2}+m_{1}^{2}m_{2}^{2}+n_{1}^{2}n_{2}^{2}=1/6$ can be proved by using an additional condition that $l_{1}l_{2}+m_{1}m_{2}+n_{1}n_{2}=0$. As a result, the Poisson ratio as well as the shear modulus in the (111) plane are also irrelevant to the crystal directions. Therefore, the in-plane elasticity of the (111) wafer can be regarded as isotropic. It might be predicted that the frequency splits of the simulation and test of the (111) VRG would be very tiny, even zero. According to the above methodology, the orthotropic elasticity values of the (100) and (111) wafers are calculated and listed in Table 1, and these values could be used to simulate resonant frequencies of a VRG.

## 3. Working Principle and Modal Simulation of VRG

### 3.1. Working Principle

The working principle of the VRG is based on the wineglass mode. The vibrating pattern of the n=2 mode is elliptical with four nodes, and two orthogonal modes are 45 degrees apart from each other. When the gyroscope is being driven at the n=2 mode, once it is under the rotation, the generated Coriolis acceleration will force the energy to transfer between two orthogonal modes [22]. The angle of rotation of the VRG can be measured by two methods. The first method, called the whole angle pattern, utilizes the inertia of the vibrating modes to measure the angle of rotation directly [23]. In the rotation, the rotation of the vibrating mode lags behind that of the case, and the lagging angle is called the precession angle [24]. The precession angle is obtained by the product of the angle of rotation and the angular gain $A_{g}$. It is noticed that the angular gain is a fixed value that only depends on the geometry and the vibrating mode of a VRG, and has nothing to do with the lifetime, etc. [25]. The angular gain $A_{g}$ defined as γ/nM is also the scale factor of the VRG used as a rate-integrating gyroscope [3]. Here, γ is the Coriolis mass, and *M* is the effective mass. The other method, named the force-to-rebalance pattern, can be used to measure the angle rate. In this pattern, the amplitude of the sense mode is driven to zero by control electrodes while the vibration of the VRG is only aligned with the drive mode, and then the angle rate can be figured out because the force produced by the control electrodes is proportional to the angle rate [22].

### 3.2. Modal Simulation

**Figure 2:**
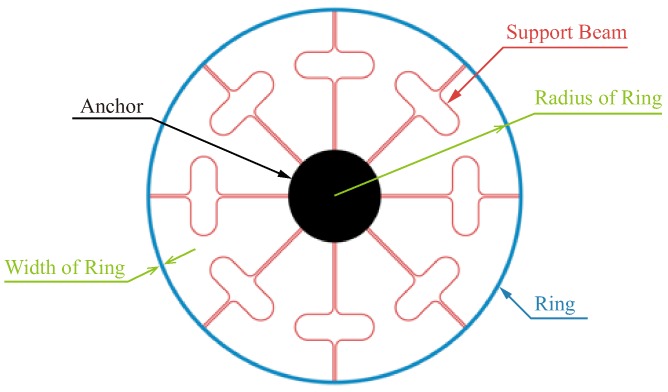
The schematic of the vibrating ring gyroscope (VRG).

The VGR simulated and fabricated in this paper is based on the structure that has been designed and reported in Reference [26]. The VRG shown in Figure 2 consists of three elements: a ring, eight support beams, and an anchor.

Resonant frequencies of the n=2 mode of the circular ring structure are crucial to those of the VRG, so it is necessary to simulate the effects of structural parameters of the circular ring on resonant frequencies and the frequency split of the n=2 mode by COMSOL (v5.4). Since the thickness of the circular ring almost does not affect the resonant frequencies of the n=2 mode, only the width and the radius are considered. In order to simulate the resonant frequencies of the circular ring of the (100) wafer, the **Anisotropic** option and the **Voigt** option are chosen in the lists of the **Solid model** section and the **Material data ordering** section, respectively, and the matrix in Equation (5) is typed in the table of **Elasticity matrix** in the **Settings** window for **Linear Elastic Material** in the interface of COMSOL. When it comes to the (111) wafer, the procedure is the same, but the matrix is changed to Equation (6).

#### 3.2.1. Width of Circular Ring

**Figure 3:**
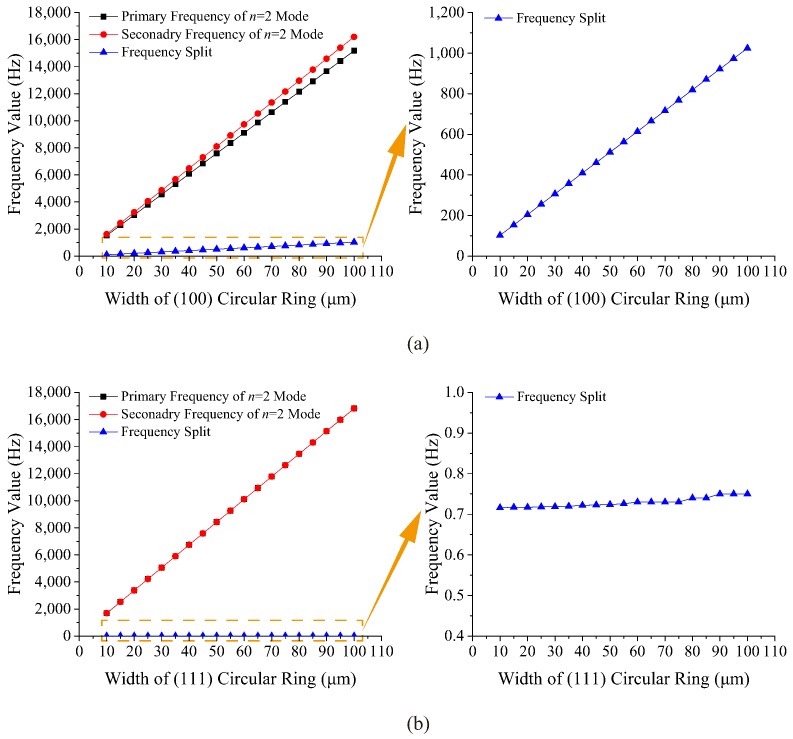
Resonant frequencies and frequency splits of rings with different widths: (**a**) (100) ring, (**b**) (111) ring.

The width plays an important role in the resonant frequencies of the circular ring because it contributes to the mass and the stiffness of the ring. The radius and thickness of the ring are fixed at 2500 μm and 120 μm, respectively, while the width distribution is from 10 μm to 100 μm, and the interval is 5 μm. Simulation results of different widths of (100) and (111) circular rings are shown in Figure 3.

It can be seen that the resonant frequencies of two types of rings increase linearly with the increase in width. The frequency split of the n=2 mode of the (100) ring caused by the anisotropy is large, and it increases linearly as the width increases. However, the primary frequency of the n=2 mode of the (111) circular ring is always almost the same as the secondary frequency, which means the frequency split of the (111) ring is very tiny and irrelevant to the width.

#### 3.2.2. Radius of Circular Ring

**Figure 4:**
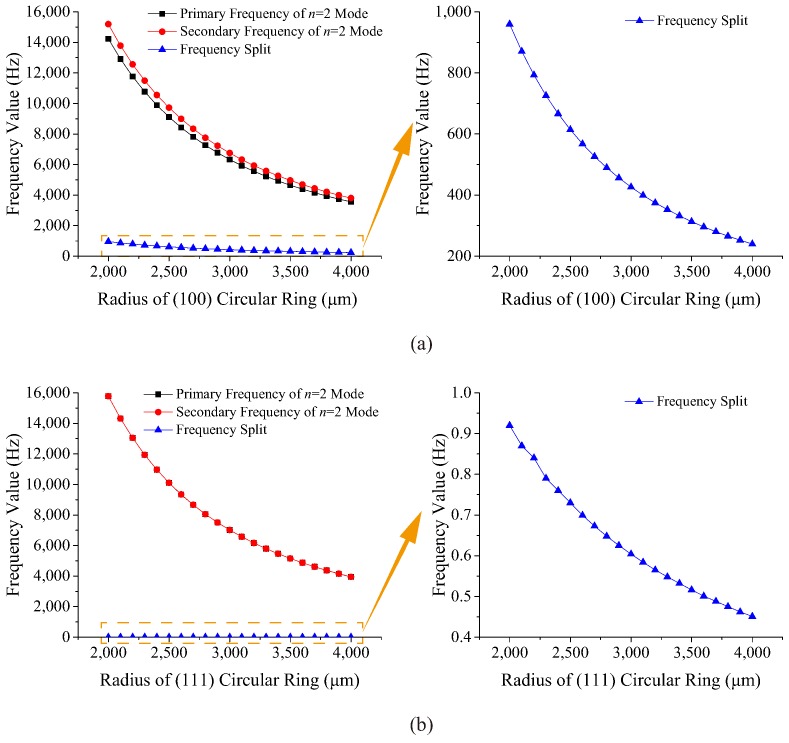
Resonant frequencies and frequency splits of rings with different radii: (**a**) (100) ring, (**b**) (111) ring.

Radius is the other factor that significantly impacts the frequency characters of the circular ring. In this case, the radius ranges from 2000 μm to 4000 μm with 21 points while the width remains at 60 μm, and the thickness remains at 120 μm. The resonant frequencies and frequency splits of two kinds of rings with different radii are shown in Figure 4.

As shown in Figure 4, the resonant frequencies of the (100) and (111) circular rings decrease as parabolic curves when the radius increases. It can be seen that the difference between the two resonant frequencies of the (100) ring drops obviously with the increment of the radius. However, the two curves of resonant frequencies of the (111) ring almost overlap, which means the frequency split is quite small.

According to the simulation above, the resonant frequencies of the (111) circular ring are always larger than those of the (100) circular ring at the same size, which indicates that the global in-plane stiffness of the (111) wafer is larger. Besides, the simulation results show that the frequency split of the (100) ring caused by the anisotropy is obvious, and it has a strong relationship with the geometry of the (100) ring while the (111) wafer is in-plane isotropic.

**Figure 5:**
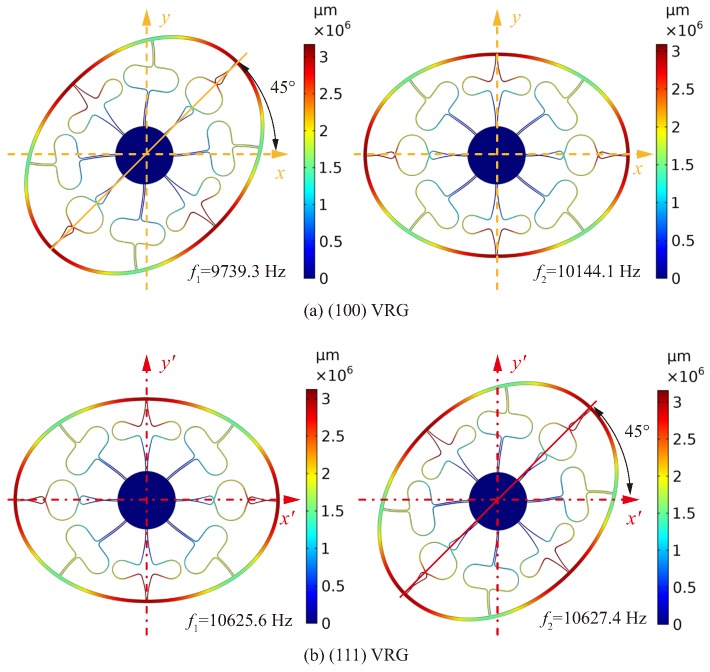
Modal shapes and resonant frequencies of (100) and (111) VRGs.

In order to further study effects of different elastic properties of (100) and (111) wafers on the resonant frequencies of the VRG, a simulation of a VRG with specific structural parameters was performed. The resonant frequencies of the VRG are set at around 10 kHz, so the final sizes of the VRG are determined after taking into account the simulation results of the circular rings and some important structural parameters as shown in Table 2. According to the simulation using stiffness matrices, modal shapes and resonant frequencies of (100) and (111) VRGs are shown in Figure 5.

According to Figure 5, the primary frequency $f_{1}$ of the n=2 mode of the (100) VRG is 9739.3 Hz, and the secondary frequency $f_{2}$ is 10,144.1 Hz, so the frequency split Δ$f_{\left(100\right)}$ between the two resonant frequencies is 404.8 Hz. The lower resonant frequency $f_{1}$ of the (111) VRG is 10,625.6 Hz while the higher resonant frequency $f_{2}$ is 10,627.4 Hz, and thus the frequency split Δ$f_{\left(111\right)}$ is 1.8 Hz. In summary, the (111) VRG has a few higher n=2 mode resonant frequencies, and its frequency split is much smaller than that of the (100) VRG, which is consistent with the theory of elastic properties of the two silicon wafers and the simulation results of the circular ring. Then, it was verified by fabrication and the test.

## 4. Fabrication and Test

The fabrication technology of the VRG is deep dry silicon on glass (DDSOG) process, which is a kind of mature bulk micromachining [27]. The silicon-glass bonding of this technology has the advantages of being a simple process, with small parasitic capacitance, and less structural stress.

**Figure 6:**
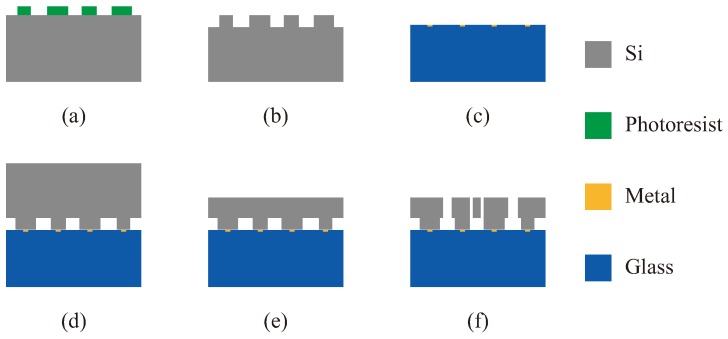
The fabrication process of VRG: (**a**) Photo-etching, (**b**) Bonding area etching, (**c**) Metal deposition, (**d**) Anodic bonding, (**e**) Thinning and polishing, (**f**) Dry etching and structure release.

The fabrication process is shown in Figure 6: (a) the photoresist is laid and photo-etched on the silicon wafer; (b) the bonding area of the silicon is formed by dry etching; (c) the metal is deposited in small grooves which are processed in the glass by wet etching; (d) the silicon wafer is bonded to the glass substrate by anodic bonding; (e) the silicon wafer is thinned and polished; (f) the structure of the VRG, of which the dimensions are shown in Table 2, is deeply etched by dry etching and then released.

**Figure 7:**
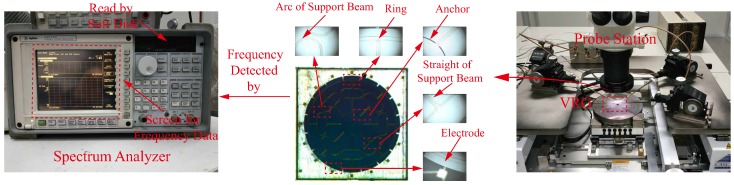
Microscope image of the VRG on the probe station.

**Figure 8:**
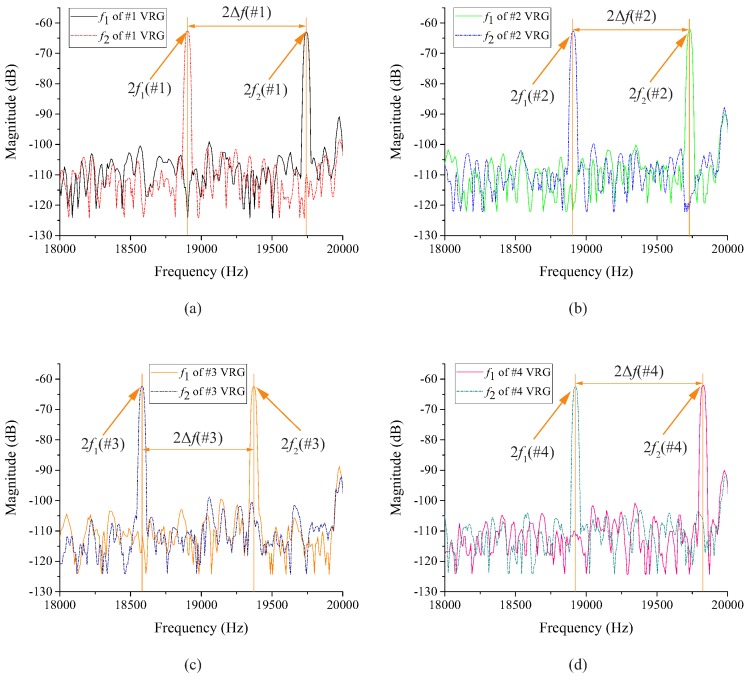
Second harmonic frequency data of four (100) VRG samples: (**a**) #1 VRG, (**b**) #2 VRG, (**c**) #3 VRG, (**d**) #4 VRG.

**Figure 9:**
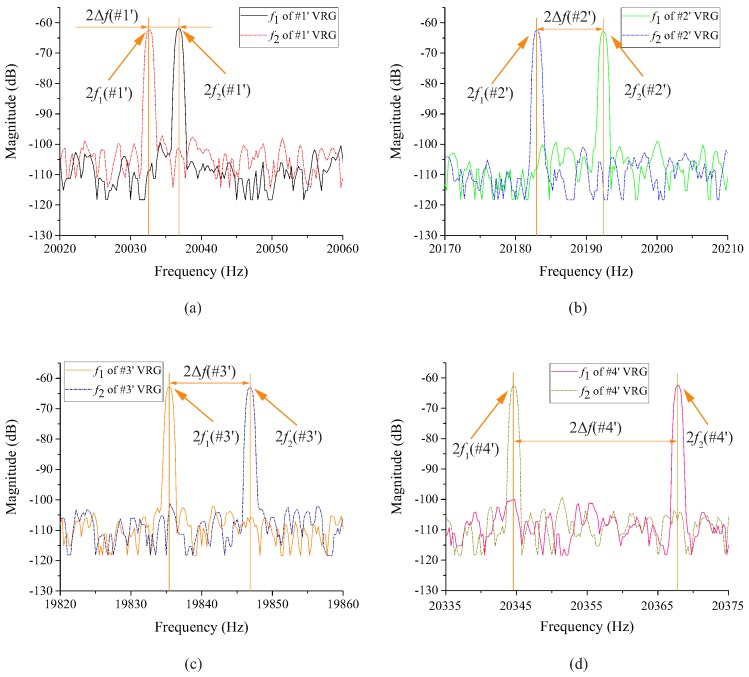
Second harmonic frequency data of four (111) VRG samples: (**a**) #1′ VRG, (**b**) #2′ VRG, (**c**) #3′ VRG, (**d**) #4′ VRG.

After the fabrication, the structure of one VRG under the microscope is shown in the middle of Figure 7. The VRGs tested on the probe station in the picture were excited by applying an 8 V DC and a 6 V AC. A signal generator was used to produce sinusoidal signals with different frequencies, then resonant frequencies ($f_{1}$, $f_{2}$) of n=2 mode of each VRG were obtained through the spectrum analyzer shown in Figure 7 by acquiring the peak value of second harmonic frequencies [28]. Besides, the amplitude-frequency curve which is displayed on the screen of the spectrum analyzer can be recorded by the soft disk. Figure 8 shows the tested second harmonic frequency data of four (100) VRG samples (#1, #2, #3, #4) while the frequency data of four (111) VRG samples (#1′, #2′, #3′, #4′) are shown in Figure 9. It should be noticed that the frequency corresponding to the peak value of each curve is twice the resonant frequency in Figure 8 and Figure 9, so the difference between the two frequencies of one VRG is also double. The specific resonant frequency data of the (100) and (111) VRGs read from the signal generator as well as calculation results are represented in Table 3, where |Δf| is the absolute value of $f_{1}$-$f_{2}$ while $|\bar{\Delta f}|$ and σ(|Δf|) are the average and standard deviation values of the absolute values.

According to test results, practical resonant frequencies and frequency splits of the (100) and (111) VRGs are close to the simulation results. Existing differences between the test data and simulation results are mainly caused by over-etching in the fabrication. Over-etching usually reduces the widths of support beams and the circular ring, which leads to a decline in the resonant frequencies. Besides, fabricated (100) VRGs generally have very large frequency splits, and the average value is 419.6 Hz while frequency splits of fabricated (111) VRGs are around 5.9 Hz. It indicates that the frequency splits of the (100) VRGs are strongly affected by the anisotropy of the (100) wafer while the small frequency splits of the (111) VRGs benefit from the in-plane isotropy; only from this point of view, is the (111) silicon wafer the preferred material for the VRG.

## 5. Conclusions

In the field of MEMS, SCS is widely used to fabricate high respect ratio VRGs along with being used in bulk silicon micromachining techniques; this is because of its outstanding mechanical and electrical properties. However, the anisotropy of SCS causes the elastic properties of the resonator to vary depending on the crystal directions, which influences the frequency characteristic of the SCS VRG. This paper presents how the anisotropy of SCS affects the frequency splits of VRGs, which can provide a method to calculate the elastic properties of various SCS wafers. Furthermore, this paper offers a basis with which to choose a suitable material to achieve VRGs of which the drive and sense modes are well matched.

In this paper, the coordinate systems of (100) and (111) SCS wafers are obtained by rotating the default coordinate system. Then, the elastic matrices and orthotropic elasticity values of these wafers are calculated using the direction cosines of transformed coordinate systems. It is found that Young’s modulus, Poisson ratio, and the shear modulus of the (111) wafer are irrelevant to the crystal directions, so the frequency split of the (111) VRG should, in theory, be near zero. Next, resonant frequencies of the n=2 mode of (100) and (111) circular rings with different sizes are simulated, and the simulation results show that the frequency split of n=2 mode of the (100) ring is significantly influenced by the geometry, while the frequency split of the (111) ring is always very tiny as a result of the in-plane isotropy. After that, modal simulations of (100) and (111) VRGs are carried out, and frequency tests of fabricated VRGs are performed to detect the resonant frequencies on the probe station. The test results match the simulation results of the VRGs well to prove that (100) VRGs have much larger frequency splits than (111) VRGs, so the resonant frequencies of the n=2 mode of (100) VRGs are more sensitive to the crystal direction, and (111) wafer is a suitable choice for the fabrication of VRGs with restrained frequency splits.

This paper analyzes the effect of the anisotropy of SCS on the frequency splits of VRGs, which can offer a reference for analyzing the elastic properties of VRGs fabricated using various SCS wafers. Future work will include the analysis of how the anisotropy of SCS influences other characteristics of the VRG, such as the quality factor.

## Figures and Tables

**Table 1:** Orthotropic elasticity values of (100) and (111) silicon wafers.

Orthotropic Elasticity Values	(100) Wafer	(111) Wafer
$E_{x},E_{y},E_{z}$ (GPa)	169, 169, 130	169, 169, 188
$\upsilon_{yz},\upsilon_{zx},\upsilon_{xy}$	0.36, 0.28, 0.064	0.16, 0.18, 0.26
$G_{yz},G_{zx},G_{xy}$ (GPa)	79.4, 79.4, 50.9	57.8, 57.8, 66.9

**Table 2:** Partial structure parameters of VRG.

Structure Parameter	Value (μm)
Radius of Ring	3000
Width of Ring	80
Radius of Anchor	750
Width of Support Beam	20
Thickness of Ring	120

**Table 3:** Frequency test data of (100) and (111) VRGs.

	(100) VRG				(111) VRG			
	#1	#2	#3	#4	$\#1^{\prime }$	$\#2^{\prime }$	$\#3^{\prime }$	$\#4^{\prime }$
$f_{1}$ (Hz)	9871.1	9865.7	9685.4	9913.2	10,018.4	10,096.2	9917.7	10,183.4
$f_{2}$ (Hz)	9451.3	9453.6	9290.5	9461.7	10,016.3	10,091.5	9923.4	10,172.3
|Δf| (Hz)	419.8	412.1	394.9	451.5	2.1	4.7	5.7	11.1
$|\bar{\Delta f}|$ (Hz)	419.6				5.9			
σ(|Δf|) (Hz)	23.7				3.8			
